# Using Shotgun Sequencing to Describe the Changes Induced by In-Feed Zinc Oxide and Apramycin in the Microbiomes of Pigs One Week Postweaning

**DOI:** 10.1128/spectrum.01597-22

**Published:** 2022-08-11

**Authors:** Juan M. Ortiz Sanjuán, Edgar G. Manzanilla, Raúl Cabrera-Rubio, Fiona Crispie, Paul D. Cotter, Juan J. Garrido, Héctor Argüello

**Affiliations:** a Pig Development Department, Teagasc Grassland Research and Innovation Centre, Moorepark, Fermoy, County Cork, Ireland; b Grupo de Genómica y Mejora Animal, Departamento de Genética, Facultad de Veterinaria, Universidad de Córdoba, Córdoba, Spain; c School of Veterinary Medicine, University College Dublin, Dublin, Ireland; d Teagasc Food Research Centre, Moorepark, Fermoy, County Cork, Ireland; e APC Microbiome Institute, University College Cork, Cork, County Cork, Ireland; f VistaMilk SFI Research Centre, Fermoy, County Cork, Ireland; g Animal Health Department, Veterinary Faculty, Universidad de León, León, Spain; University of Nebraska-Lincoln

**Keywords:** antibiotics, diarrhea, metagenomics, pigs, swine, weaning, zinc oxide

## Abstract

Postweaning diarrhea (PWD) is a relevant problem associated with early weaning on pig farms. For decades, in-feed antibiotics and therapeutic zinc oxide (ZnO) have been widely used to prevent PWD in piglets. The European Union is banning both strategies in 2022 due to antimicrobial resistance and environmental contamination concerns, respectively. Understanding the effects of these products on the pig microbiome is crucial for correcting potential microbial disbalances that would prompt PWD. Using shotgun sequencing, three trials were carried out to explore the impact of in-feed apramycin and ZnO, combined with different farm hygiene protocols, on the fecal microbiomes of piglets 7 days postweaning. In trial 1, 28-day-old piglets were allocated to one of three groups: control diet (Ct), Ct + ZnO (Zn), and Ct + apramycin (Ab). In trials 2 and 3, piglets were allocated to the same treatments, but the trials also included different cleaning protocols, achieving different hygiene levels. In-feed treatments impacted the richness, diversity, and relative abundance of the piglets’ microbiome more than hygiene. Pigs in the Ct group showed higher species richness than pigs in the Ab and Zn groups. A clustering analysis evidenced a link between *Enterobacteriaceae* in the Ct group; *Lactobacillaceae* and *Veillonellaceae* mainly in the Ct group; and *Bacteroidaceae*, *Ruminococcaceae*, *Oscillospiraceae*, *Acidaminococcaceae*, and *Lactobacillaceae* in the Ab and Zn groups. Functional data analysis revealed a higher abundance of virulence genes in the Ct group microbiomes and heavy metal and antimicrobial resistance-related functions in the Zn treatment group. The results demonstrate that alternatives to Ab and ZnO should balance the microbial abundance and stimulate the growth of commensals to outcompete potential pathogens.

**IMPORTANCE** Weaning is a critical period for piglets, during which potentially harmful bacteria such as Escherichia coli can increase in abundance in the intestine, creating digestive problems and diarrhea. In-feed antibiotics, the most frequent administration route for antibiotics in livestock, and therapeutic doses of zinc oxide (ZnO) help to control diarrhea but prompt secondary problems such as antimicrobial resistance and soil pollution from heavy metals. Understanding how these strategies impact the gut microbiota is crucial for establishing health biomarkers and designing successful replacement strategies. Using shotgun sequencing, this study compares the microbiota of pigs after early weaning when treated with in-feed antibiotics, ZnO, or treatment-free diets to describe differences that could define the susceptibility to infections, providing the basis for future research on improving intestinal resilience through microbiota-based strategies.

## INTRODUCTION

Weaning is a critical period, during which piglets are abruptly exposed to stressors such as an early separation from the mother, a sudden dietary change from milk to solid feed, and/or transport to a new environment where they are mixed with other litters (1). Two major consequences of weaning are the piglets becoming immunocompromised (2) and a transient gut dysbiosis promoted by the sudden nutrient changes (3). This is an opportunity for pathogens to colonize the gut and establish an infection, leading frequently to postweaning diarrhea (PWD), a multifactorial disease causing economic and productive losses on pig farms. PWD usually involves the presence of enterotoxigenic Escherichia coli (ETEC) and requires antimicrobial therapies to control the problem (4, 5).

Within the context of enteric diseases, the commensal gut microbiota can play a pivotal role, acting as an additional protective layer by competing with pathogens for niches in the intestine, a concept known as colonization resistance (6). Indeed, early establishment of a desirable microbiota after weaning can promote intestinal health and prevent enteric diseases (3, 7). The postweaning microbiota is influenced by factors such as the mother’s microbiota, the environment and cleaning protocols used (8), and in particular, diet (9). Despite this generally accepted view, the importance of each individual factor is still not well understood.

Antibiotics and therapeutic zinc oxide (ZnO) have been widely used in feed as prophylaxis and metaphylaxis to prevent and/or treat PWD. Although antibiotics directly inhibit the growth of pathogens such as ETEC, they also contribute to an increase in antimicrobial resistance (AMR), which is a major public health concern. In contrast, the mechanisms of action of ZnO are not well known. Zinc is an essential element required as a cofactor in multiple biological processes (10), and piglets may undergo a zinc deficiency at weaning (11). The effects of ZnO also include modulation of the gut morphological structure, mucus composition, and intestinal permeability (12, 13), as well as potential antimicrobial impacts (14, 15). Altogether, the use of ZnO results in reduced inflammation and diarrhea (16, 17) and improves growth performance (18). However, the high dosages of therapeutic ZnO (over 2,000 ppm) required and its poor absorption in the gut are a major cause of environmental pollution of soil where pig slurry is used as a fertilizer (19). Due to these concerns, ZnO and antimicrobial prophylactic medication will be banned in the European Union starting in 2022 (20), and other countries/regions have introduced or will soon introduce similar regulations.

By studying the modulation of the intestinal microbiota by ZnO and antibiotics, the potential exists to gain insights into the key changes that contribute to the control of PWD with a view to identifying other microbiome modulators than could be employed in the forthcoming post-ZnO and AB-free era. Thus, the present study used shotgun sequencing to determine the impact of in-feed apramycin and therapeutic in-feed ZnO on the fecal microbiomes of early weaned pigs, while also assessing the relative importance of the background hygiene protocols in the facilities.

## RESULTS

Three trials were carried out to assess the fecal microbiomes of pigs supplemented with an in-feed antibiotic (apramycin) (Ab group) or in-feed ZnO (Zn group) relative to a control (Ct) group. The productive performance of the animals was not affected by the in-feed treatments or cleaning protocols in any of the three trials, although pigs in the Ct group exhibited softer feces. The average weight gain was 193, 169, and 173 g/day, and the average feed intake was 240, 184, and 215 g/day for trials 1, 2, and 3, respectively, resulting in feed conversion rates of 1.37, 1.31, and 1.33.

All data related to the microbiome were analyzed from an overall global perspective as well as for each individual trial, considering the cleaning protocols used in each trial. Trial 1 only had standard cleaning. Trial 2 included two cleaning protocols (Table 1), a substandard protocol to study the effects of deficient hygiene and an optimal cleaning protocol to study the effects of excellent hygiene. Dietary treatments had a much more marked effect on the microbiome than cleaning protocols. Trial 3 included a group of pigs with no cleaning protocol and a group with a substandard cleaning protocol to study the effects of deficient hygiene compared to a standard protocol (Table 1). Again, most effects observed were a consequence of dietary treatments and not of the cleaning protocols.

**TABLE 1:** Cleaning protocols used in the three trials

Trial	Cleaning protocol		Presoak^*a*^	Detergent^*b*^	Power wash^*c*^	Disinfectant^*d*^	Dry
	Code	Description					
1	A	Standard	Yes		Yes	Yes	Yes
2	B	Substandard			Yes	Yes	
2	C	Optimal	Yes	Yes	Yes	Yes	Yes
3	D	No cleaning					
3	E	Substandard	Yes		Yes		Yes
3	F	Standard	Yes		Yes	Yes	Yes

a Presoak: rooms were equipped with sprinklers in the ceiling. Sprinklers with cold water (10 to 15°C) were turned on for approximately 1 h 40 min. Then, the room was power washed.

b Detergent: kenosan (CID Lines, Belgium) used at 0.5% was applied to all surfaces in the rooms for approximately 1 h 30 min after presoaking. Then, the rooms were power washed.

c Power wash: cold water (10 to 15°C) was applied using a power hose to all surfaces in the room. The rooms were then left to dry for at least 24 h.

d Disinfectant: after washing, Hyperox (Virkon, used at 1%; LANXESS Deutschland GmbH, Germany) was applied to all surfaces in the rooms. The rooms were then left to dry for at least 48 h.

### In-feed treatment with ZnO or apramycin limits species richness at weaning.

**FIG 1:**
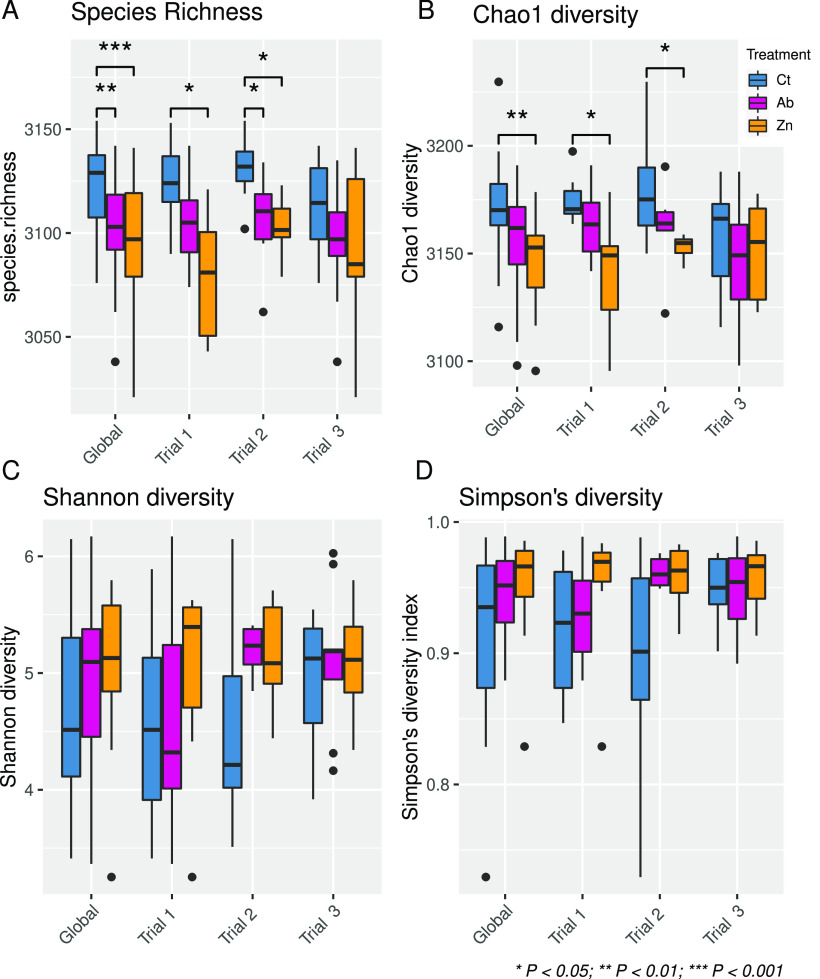
Alpha diversity richness—species richness (A), Chao1 diversity index (B), and Shannon diversity index (C)—and evenness (Simpson diversity index) (D) of samples from different dietary treatments. The information is provided for the global analysis and trials 1, 2, and 3. The lower, middle, and upper horizontal lines in the boxes correspond to the first, second, and third quartiles (the 25th, 50th, and 75th percentiles). The lines extending above and below the boxes indicate the range of the upper and lower points within the 1.5 interquartile range. Taxonomic identification of sequences was performed using Kraken v2.

The outputs from the microbial richness (species richness, Chao1 and Shannon indexes) and evenness (Simpson index) analyses are summarized in Fig. 1 (for further details, see File S1 in the supplemental material). The global analysis revealed a higher species richness in the feces of the Ct group pigs compared to pigs in the Ab (*P = *0.003) or Zn (*P < *0.001) treatment groups (Fig. 1A), while the Chao1 diversity values were also higher in the control group compared to the Zn-treated pigs (*P = *0.002) (Fig. 1B). No differences among the groups were observed for the Shannon or Simpson indexes (Fig. 1C and D).

The differences in the global analysis were consistent for trial 1 and trial 2, where samples from the control pigs had greater species richness (trial 1, *P = *0.007; trial 2, *P* = 0.019) and Chao1 diversity (trial 1, *P = *0.007; trial 2, *P = *0.043) than those from the Zn group pigs, as well as greater richness than samples from the Ab group in trial 2 (*P = *0.033). No differences were observed among dietary treatments in trial 3 or among cleaning protocols in trials 2 or 3 (Fig. S1 in the supplemental material).

### Microbiota ordination is impacted by in-feed treatments rather than environmental cleaning.

**FIG 2:**
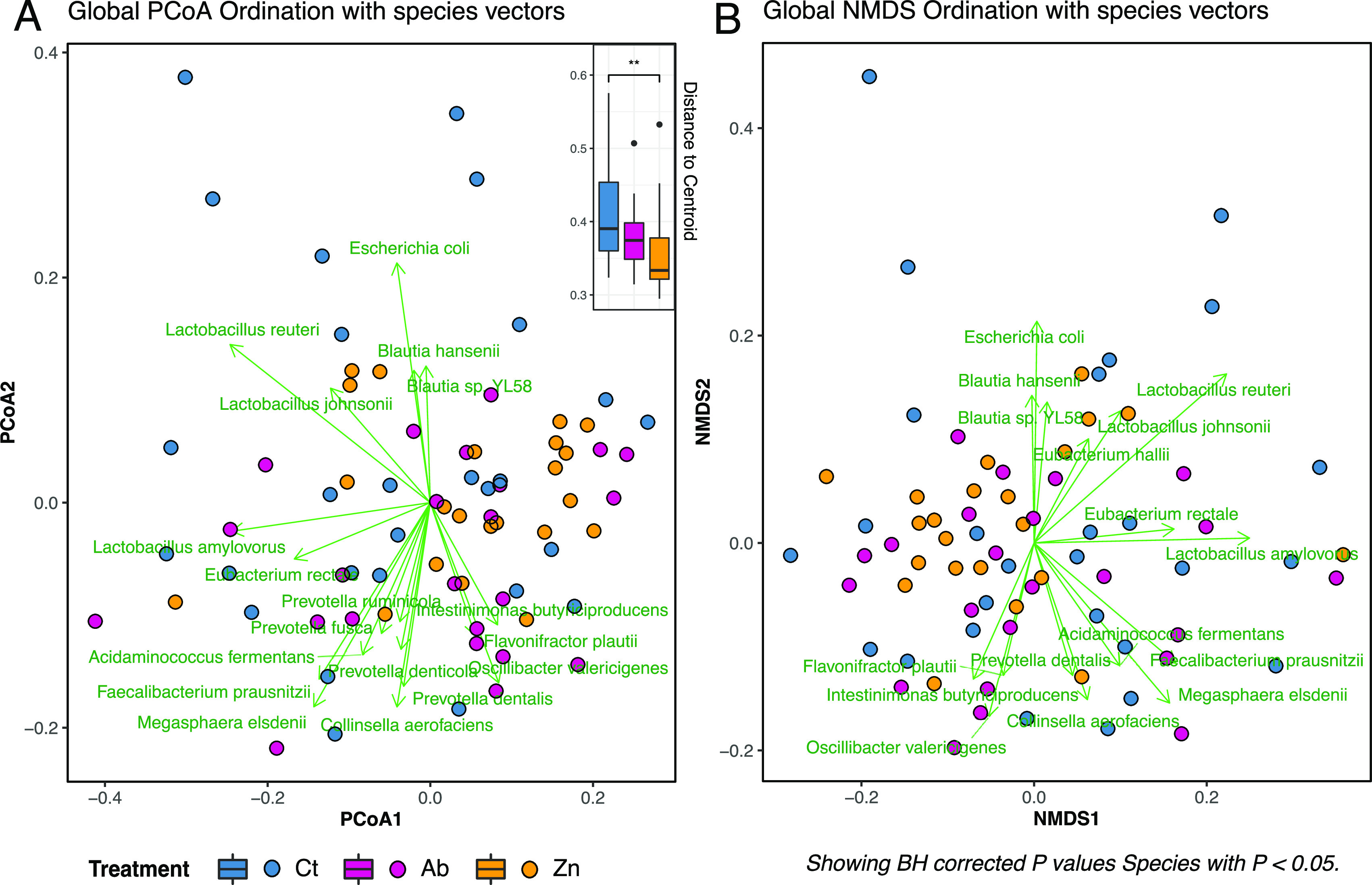
Ordination analyses of fecal samples from weaned pigs with different dietary treatments (control, apramycin, and ZnO). (A) PCoA ordination of the global analysis of fecal samples, along with a boxplot of the within-group dispersion analysis of each dietary treatment. **, *P < *0.01. (B) Nonmetric multidimensional scaling (NMDS) ordination of the global analysis. Panels A and B show the significant species returned by the envfit model. (Arrows indicate the species with significant Benjamini-Hochberg [BH]-adjusted *P* values; the length of the arrows represents the strength of each species’ influence on the ordination of samples). Taxonomic identification of sequences was performed using Kraken v2.

Analysis of the within-group dispersion revealed differences between the control and ZnO treatment groups (Fig. 2A). The influence of dietary treatments on the global analysis ordination was confirmed by the envfit function when visualized by principal coordinates analysis (PCoA) (Table S1) (*P = *0.041). The sample ordination was clearly influenced by the abundance of species, as shown in Fig. 2A and B.

Permutational multivariate analyses of variance (PERMANOVA) revealed differences in the dietary treatment variable (Table S2) (*P = *0.001). Further analyses using pairwise PERMANOVA depicted the differences among all dietary treatment levels (Table S2) (*P < *0.01).

Ordination of the samples and PERMANOVA of the respective trials revealed no differences among the trial 1 treatments (Table S1 and S2; Fig. S2A and S2D). In trial 2, microbiomes from the Ct diet pigs showed higher dispersion than the Ab and Zn dietary treatment pigs (Fig. S2E); and ordination and PERMANOVA revealed differences among the cleaning and dietary treatments (Table S1 and S2; Fig. S2B and S2E). In trial 3, ordination and PERMANOVA revealed differences between Zn and the other two dietary treatments (Table S1 and S2; Fig. S2C and S2F).

### Dietary treatments shape the microbiota composition and impact the dominant core species.

**FIG 3:**
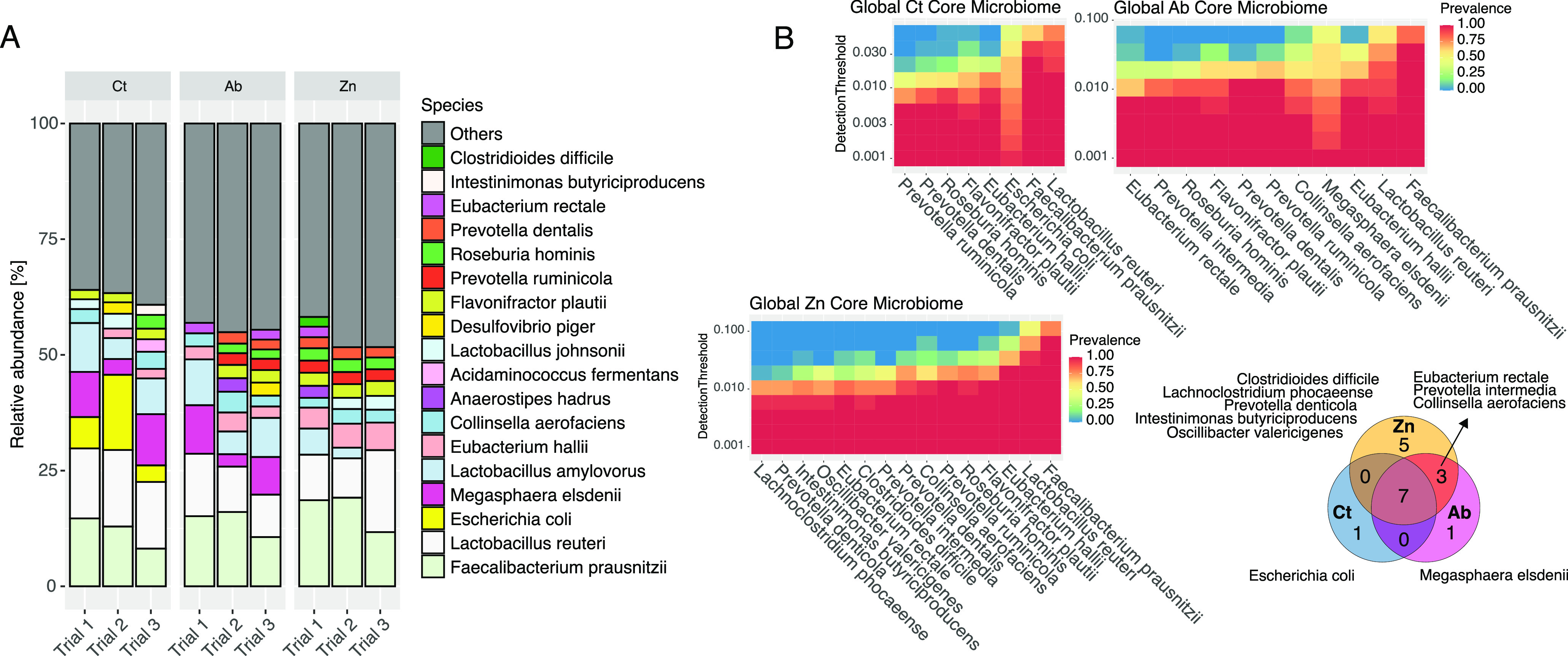
Fecal microbiota composition in weaned pigs with different dietary treatments (control, apramycin, and ZnO). (A) Mean relative abundance of the main species in each group and trial. “Others” refers to those species accounting for less than 2% abundance. (B) Core microbiome of pigs for each dietary treatment. The inclusion criteria for species were an abundance detection threshold of 1% in at least 70% of the samples of each group. Taxonomic identification of sequences was performed using Kraken v2.

The microbiota abundance was dominated by 18 species that accounted for approximately 60% of the fecal relative abundance (Fig. 3A). Faecalibacterium prausnitzii and Lactobacillus reuteri were consistently the dominant species, regardless of the treatment group (Fig. 3A; File S2). Other dominant species were Megasphaera elsdenii and Lactobacillus amylovorus, among others (File S2). Most of these species defined the core microbiota of each group (Fig. 3B).

**FIG 4:**
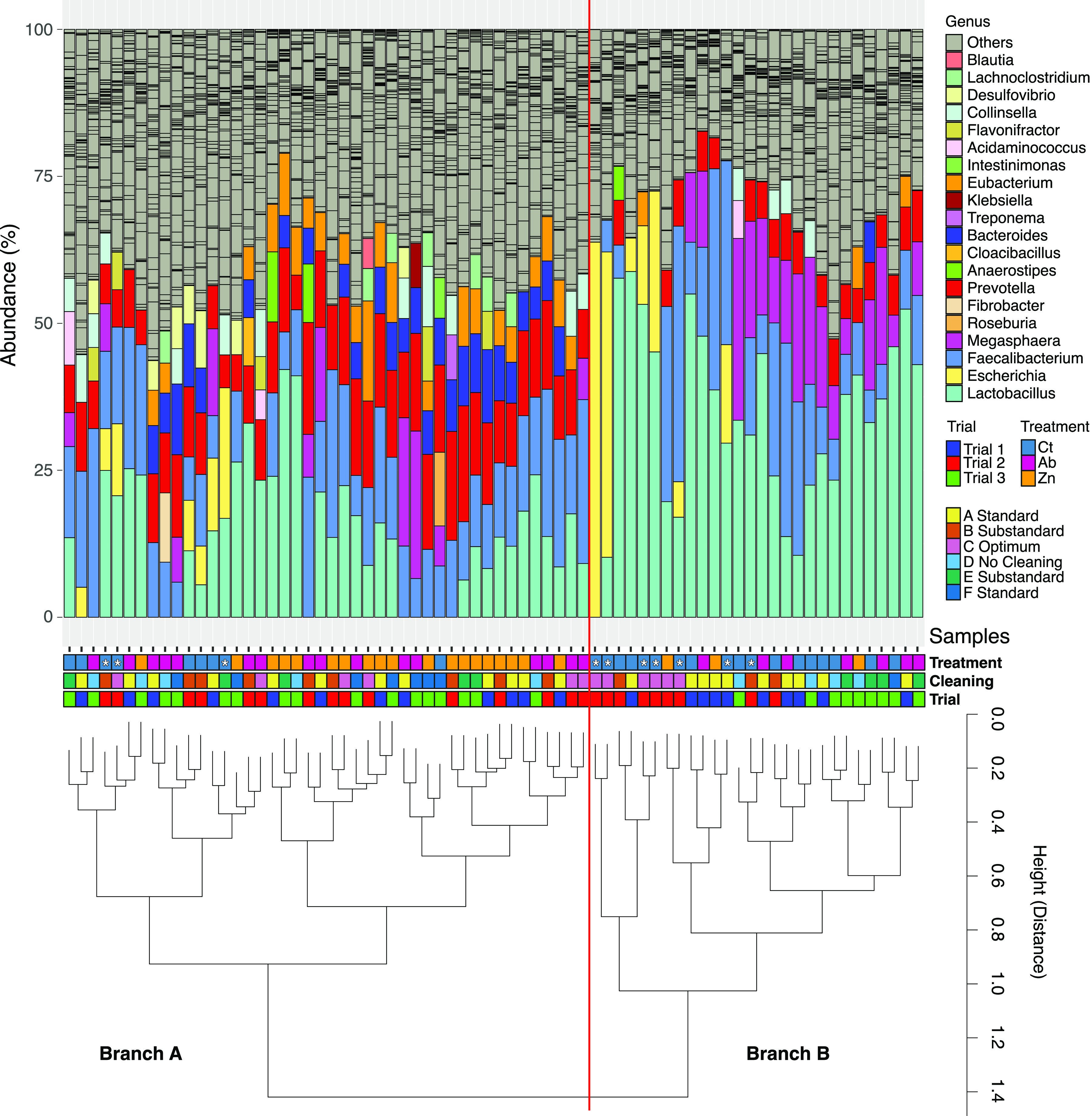
Stacked bar plot of the relative abundance of the main genera in fecal samples from weaned pigs with different dietary treatments. Profiles of samples (top) are ordered by Ward clustering of the squared Bray-Curtis distances between samples. The cluster dendrogram (bottom) represents the similarity between the microbial composition of the samples. Variables for each sample (from lower to upper, trial number, cleaning protocol, and dietary treatment) are indicated in the colored squares below the bars. For better visualization, a red vertical line divides the relative abundance profiles into the two patterns established by the Ward clustering. “Others” refers to those genera accounting for less than 5% abundance. Taxonomic identification of sequences was performed using Kraken v2.

The abundance of different species clearly grouped the samples by in-feed treatment using Ward clustering and Bray-Curtis distances. Thus, 18 out of 27 control samples clearly clustered in one of the two branches comprising 28 pigs (Fig. 4, branch B), dominated by the high relative abundance of Escherichia coli, *Lactobacillus* spp., *Faecalibacterium* spp., and *Megasphaera* spp. Indeed, core microbiome analyses, performed at an abundance threshold of 1% of the total abundance in 70% of the samples of each group, identified E. coli as the singular taxon in the core microbiota of control animals (Fig. 3B). The other branch (branch A) in Fig. 4 included mainly pigs from the Ab and Zn treatment groups (total, 35 of 44 pigs; branch A), where the most representative species were *Lactobacillus* spp., *F. prausnitzii*, *Bacteroides* spp., or *Prevotella* spp., which accounted, in most cases, for approximately 50% of the relative abundance.

**FIG 5:**
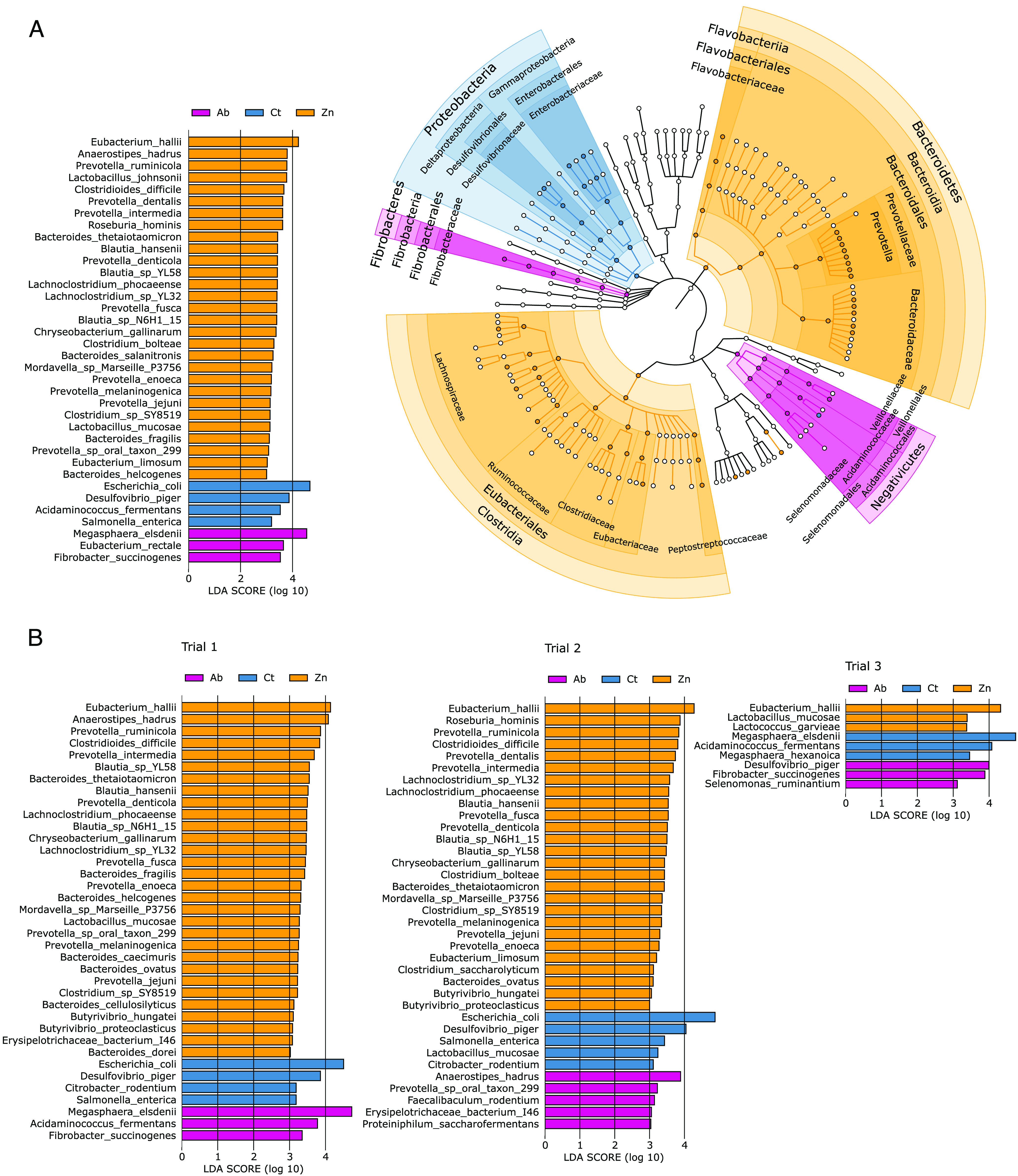
Differences in species abundance, returned by linear discriminant analysis effect size (LEfSe) analysis, most likely explaining the differences among dietary treatments. (A) Global analysis: species (left) and taxa (right) associated with each dietary treatment. (B) Differences in species abundance, returned by the LEfSe analysis, most likely explaining the differences among dietary treatments in trials 1, 2, and 3. The horizontal bars are colored according to the dietary treatment and represent the effect size of each species in each particular group. The background shades and clades (circles) of the cladogram (panel A, right) are colored according to the associated dietary treatment found by LEfSe.

A cladogram of bacterial groups linked to any of the treatments by linear discriminating score analysis (LDA) evidenced an association of *Clostridia* and *Bacteroides* with the Zn group, *Negativicutes* and *Fibrobacteres* with the Ab group, and *Proteobacteria* with the Ct group (Fig. 5A). Differences in species abundance as determined by linear discriminating score analysis again revealed a higher abundance of E. coli, Desulfovibrio piger, Acidaminococcus fermentans, and Salmonella enterica in the Ct group pigs, while *M. elsdenii*, Eubacterium rectale, and Fibrobacter succinogenes were more abundant in the Ab animals. The Zn animals were enriched in species belonging to the following families: *Bacteroidaceae*, *Prevotellaceae*, *Flavobacteriaceae*, *Clostridiaceae*, *Eubacteriaceae*, *Lachnospiraceae*, *Peptostreptococcaceae*, and *Ruminococcaceae* (Fig. 5A). The singular species in the Zn diet core microbiome were represented by Clostridioides difficile, Lachnoclostridium phocaeense, Prevotella denticola, Intestinimonas butyriciproducens, and Oscillibacter valericigenes. Species found uniquely at the established levels of abundance and prevalence in the Ct and Ab animals were E. coli and *M. elsdenii*, respectively. The species E. rectale, Prevotella intermedia, and Collinsella aerofaciens were shared between the Ab and Zn core microbiomes (Fig. 3B).

Similarly, analysis of the abundance by trial (Fig. 5B) revealed a systematically higher abundance of E. coli in the Ct animals, as well as other *Proteobacteria* (*D. piger*). A higher abundance of *F. succinogenes* was linked to Ab animals in trials 1 and 3. We also observed a link between Zn treatment and a consistently higher abundance of Eubacterium hallii in the 3 trials; we noted the presence of different species belonging to the families *Bacteroidaceae*, *Prevotellaceae*, and *Lachnospiraceae* in trials 1 and 2, while Lactobacillus mucosae was higher in abundance in the Zn animals in trial 1 and trial 3, and Lactococcus garvieae in trial 3. *M. elsdenii* was higher in abundance in the Ab animals in trial 1 and the control pigs in trial 3, showing inconsistent results. As well, different species changed groups; for example, Anaerostipes hadrus shifted from the Zn to Ab groups in trials 1 and 2, and *A. fermentans* shifted between the Ab and Ct groups in trials 1 and 3.

Finally, we obtained 138 metagenome-assembled genomes (MAGs) from 61 species (Fig. S3). The MAGs of some species were ascribed to particular dietary treatment groups. Thus, the seven E. coli MAGs were present in the control samples, while 13 *Prevotella* spp. were linked to 6 samples from Ab animals, 6 samples from Zn animals, and 1 from a Ct animal, this last genome clustering in a different group.

### ZnO and apramycin increase the gene functions associated with metabolism, while treatment-free diets increase virulence factors.

Figure S4 summarizes the microbial functions by the differentially abundant genes identified in each dietary medication group. Global analysis and analysis by trial highlighted a higher abundance of genes associated with carbohydrate metabolism in feces from the Zn-supplemented pigs. These animals exhibited higher abundance pathways associated with the metabolism of mono-, di-, oligo-, and polysaccharides. The Ab group had few differentially abundant functions related to protein metabolism, pyrimidines, or amino acid assimilation (Fig. S5E), while animals in the Ct group exhibited a higher abundance of functions associated with virulence, such as siderophores, oxidative stress, types III, IV, and VI secretion systems (Fig. S4F), and other virulence factors (Fig. S4J).

## DISCUSSION

The use of in-feed medications such as antibiotics and ZnO have supported the control of PWD over the last few decades. Among the potential antibiotics to treat PWD, apramycin is indicated to treat porcine colibacillosis and has been widely used since the 1980s (4, 21). It is a bactericidal antibiotic within the group of aminoglycosides that inhibits bacterial protein synthesis by irreversibly binding to the receptor proteins on the 30S subunit of the bacterial ribosome (22). Both treatments not only directly inhibit the growth of pathogens but also modulate the piglets’ gut microbiota composition (23, 24). However, more knowledge is needed about the precise impact of these medications on the microbiota in order to clarify which bacteria and functional roles are misplaced when these treatments are removed. This information is of paramount relevance for designing strategies to successfully replace antibiotics and ZnO in PWD control. In this study, we used shotgun metagenomics to explore the changes in the fecal microbiota composition and functionality of weaned piglets arising from the use of these medications.

Alpha diversity measures the richness and diversity of the microbiota composition. We observed lower richness values in groups with in-feed medication, particularly in the Zn group. Indeed, several studies that employed 16S rRNA sequencing have reported a loss of richness in the fecal microbiota of therapeutic ZnO-treated animals (16, 17, 25). The other diversity measure, β-diversity, allows comparison of the microbiota across samples. In this study, the microbiota of the Ab and Zn groups had a higher within-group similarity than the Ct animals, which showed higher intragroup dispersion. Indeed, the lower dispersion in microbiota samples from the Zn pigs, regardless of whether the samples were collected from trials 1, 2, or 3, suggests that ZnO shapes and homogenizes the intestinal microbial composition even in different batches of pigs. Not only did the treatments shape a more homogeneous microbiota, but further abundance analyses also revealed a richer core microbiota in the Ab and Zn groups compared to the control pigs (see Fig. 3B and 4). The restrictive parameters used to calculate the species core ensured the finding of consistently present species in each group, thereby removing rare species and keeping the dominant ones (which are usually the well-known microorganisms). Ward clustering allowed us to clearly differentiate samples into two major branches (Fig. 4A and B). Branch B was formed mainly by the control animals and was dominated by E. coli, *Megasphaera* spp., *F. prausnitzii*, and *Lactobacillus* spp. Branch A included mainly pigs from the Ab- and Zn-treated groups and was dominated by a more diverse pattern of abundance of the genera *Eubacterium*, *Bacteroides*, *Prevotella*, *F. prausnitzii*, and *Lactobacillus* (altogether about 60% of the fecal relative abundance) and in which E. coli accounted for less than 5% of the relative abundance. In this study, animals did not show clinical signs of PWD, and there were no significant differences in productive performance among the treatments. However, piglets on the Ct diet were the only ones where softer feces were observed at sampling. Samples showing a softer consistency are annotated with an asterisk in Fig. 4. Not all soft feces had a high abundance of E. coli, but most of them were associated with branch B (Fig. 4). E. coli is the principal pathogen involved in PWD (3), and in-feed medication, both ZnO and antibiotics, aims to reduce the growth of this bacteria in the postweaned piglet gut (26, 27). Accordingly, the removal of in-feed medications resulted in an increase of E. coli abundance and virulence functions related to secretion systems, siderophores, hemolysins, and fimbriae in the microbiota of the nonmedicated pigs. These functional profiling results reveal different mechanisms linked to colonization and pathogen survival and demonstrate that potential functional biomarkers of intestinal disbalance or gut dysbiosis can be obtained in functional analyses of the microbiome.

Similar to previous studies (28), overgrowth of E. coli was linked to the rise of other *Proteobacteria* such as *D. piger* in control samples, a bacteria linked to unhealthy enteric status (29) and which seems to be controlled by ZnO (24). The clear association of these and other *Proteobacteria* with the Ct animals clearly evidences that either both ZnO and apramycin limited their growth or that the microbiota in these treated animals exerted a competitive exclusion against these bacterial groups.

A specific aim of this study was to shed light on which particular bacteria are influenced by these in-feed treatments; in particular, the specific impact of ZnO on the gut microbiota still remains a topic of debate. For this reason, we designed a study with three different trials, and we used shotgun metagenomics to characterize the taxonomy up to the species level. The results consistently evidenced the association of Zn treatment with species belonging to *Ruminococcus*, *Eubacterium*, *Clostridium*, *Blautia*, *Lachnoclostridium* (*Lachnoclostridium phocaeense* exclusively in the core microbiota of the Zn group), and *Roseburia*, all from the order *Eubacteriales* (phylum *Firmicutes*, class *Clostridia*). Previous studies support the presence of members of *Lachnospiraceae* in ZnO-based diets (23, 24). Members of the class *Clostridia* such as those enriched in ZnO-treated animals are considered part of the desirable anaerobic microbiota, renowned fiber-degrading bacteria and short-chain fatty acid (SCFA) producers (30, 31). Some of these functions were clearly highlighted in the functional profiles of the Zn-treated animal metagenomes. Of the taxa that were enriched in the Zn-supplemented animals, several species, such as Roseburia hominis and *L. mucosae*, have already been linked to improvement of the intestinal barrier and host immune response modulation (32, 33).

The systematic use of antibiotics in livestock both for animal treatment and as growth promoters has favored the growth of commensals that are particularly adapted to this challenging environment, exemplified by *Prevotella* spp. (28, 34, 35) and *M. elsdenii* (36–38). Both bacteria are important gut inhabitants after weaning (9), responsible for the degradation of complex saccharides and SCFA production (28, 35). Interestingly, while *Prevotella* adapted to both the Zn- and Ab-treatment environments, the growth of not only *M. elsdenii* but *Negativicutes* members in general (including *Veillonellaceae* and *Acidaminococcaceae*) was only favored by treatment with apramycin. A previous study stressed the limited ability of *Veillonellaceae* and some members of *Acidaminococcaceae* to colonize and grow in the gut with ZnO (23), and this may explain the delay in intestinal colonization by these groups after weaning. However, as stated above, these commensals seem to have adapted to exposure to different antimicrobials (28, 39).

Different cleaning protocols with variable degrees of hygiene were applied in the trials performed. Livestock is usually managed in intensive farming conditions under the so-called all-in/all-out system, in which facilities are emptied and cleaned between batches. Considering the potential effect of the environment on the establishment of the microbiota after weaning (8) and the lack of specific studies addressing it in piglets at weaning, we decided to evaluate the extent to which the cleaning protocol influenced the reshaping of the pigs’ microbiota a week after weaning. Analyses of the factors influencing microbiota ordination clearly reflected, regardless of the cleaning protocol applied or the trial, that the in-feed treatment (ZnO, apramycin, or control diet) was the factor shaping the gut microbiota. The effects of cleaning and the environment may be observed when other variables are under control. Our results clearly evidence that the potential effect is negligible compared to the influence of the in-feed medications.

The current study is one of the first addressing PWD and Zn use by shotgun metagenomics. Shotgun sequencing identifies organisms to the species level, and current analysis pipelines have improved taxonomic identification. Additionally, shotgun sequencing allows the exploration of functional profiling and microbial genomes through MAG analyses. On the other hand, its limitations are its bias toward model organisms and cultivable bacteria (which is constantly improving because of increased accuracy in databases), its inability to distinguish between live and dead microorganisms or active genes, and the compositionality problem linked to microbial data sets, which does not reflect the real absolute molecule concentration within the studied sample. Nevertheless, most of these caveats are being solved through a large number of published bacterial reference genomes, high-quality metagenomic assemblies, and new computational and statistical approaches (40).

### Conclusion.

Here, we used shotgun sequencing to offer a detailed characterization of the microbiota composition soon after weaning in piglets medicated with in-feed ZnO, antibiotics, or medication free. As occurs under real conditions on commercial farms, variability between batches is reflected across the three trials performed in this study. ZnO and apramycin modulate the microbiota composition and functionality, irrespective of the batch and cleaning protocols, enabling better bacterial carbohydrate utilization and amino acid synthesis and generating a different relative abundance pattern that tends to resemble a more mature microbiota. Future preventive measures to control PWD should involve strategies to enhance the establishment and growth of beneficial gut inhabitants that outcompete potential pathogens.

## MATERIALS AND METHODS

### Experimental design.

Three trials were carried out to study the role of in-feed ZnO and antibiotics, in combination with different cleaning protocols, on the fecal microbiota of piglets 1 week after weaning. The three experiments conducted in this study were carried out at the Teagasc Pig Research Facility and were licensed by the Teagasc Animal Ethics Committee. This research facility was established in 2016 and has since been free of the main infectious diseases affecting pig farms (i.e., porcine reproductive and respiratory syndrome [PRRS], enzootic pneumonia, pleuropneumonia, swine flu, ileitis, dysentery, edema disease, and streptococcal meningitis). The animals are regularly vaccinated for those diseases present in the herd (*Erysipelas*, parvovirus, porcine circovirus, and neonatal E. coli and *Clostridium* spp.), and therapeutic zinc oxide and in-feed antibiotics are not used regularly. Piglets were weaned at 4 weeks of age in all the trials and moved to the experimental rooms. The treatment groups were balanced by weight, and the pigs were weighed at pen level on days 0 and 7 postweaning. The rooms were equipped with fully slatted plastic floors with automatic environmental control, each pen having a single-space wet-dry feeder and a nipple drinker. The animals used in this study were Danish Duroc × (Large White × Landrace). The pigs were fed a pelleted starter diet in dry form that met commercial nutritional requirements (Table 2). Feed was provided in bags, and the intake was recorded at pen level by weighing the bags weekly. Trial 1 studied the influence of the dietary treatments (control diet [Ct], control diet + 3,000 ppm ZnO [Zn], and control diet + 100 ppm apramycin as Apralan G200 [Ab]). Trials 2 and 3 studied the same in-feed treatments in combination with different cleaning protocols (Table 1) for the rooms that were part of another study (41). A detailed description of each trial is provided further below.

**TABLE 2:** Ingredients and nutrient composition of the diet used in the three trials in this study on an as-fed basis

Dietary constituent	Amount (%)
Ingredients	
Maize	30.0
Soybean meal 48%	18.7
Whey permeate	15.0
Wheat	10.0
Full fat soy	7.00
Barley	6.74
Skimmed milk	5.00
Vegetable oil	3.85
Calcium carbonate	0.80
Dicalcium phosphate (anhydrous)	0.70
Sodium chloride	0.30
l-lysine HCl	0.672
l-threonine	0.342
dl-methionine	0.318
l-tryptophan	0.127
l-valine	0.126
Phytase (5,000 FTU/g)^*a*^	0.01
Vitamin and trace mineral mix^*b*^	0.30
Calculated composition (% as fed or as specified)	
Dry matter	89.44
Net energy (MJ/kg)	10.95
Crude protein	19.00
Standardized ileal digestible lysine	1.41
Fat	6.84
Neutral detergent fiber	8.06
Calcium	0.77
Digestible phosphorus	0.42

a FTU, phytase units.

b Provided per kg of feed: 400 mg copper sulfate, 450 mg ferrous sulfate monohydrate, 60 mg manganese oxide, 150 mg zinc oxide, 1 mg potassium iodate, 0.6 mg sodium selenite, 6 mIU vitamin A, 1 mIU vitamin D_3_, 100 mIU vitamin E, 4 mg vitamin K, 15 mg vitamin B_12_, 2 mg riboflavin, 12 mg nicotinic acid, 10 mg pantothenic acid, 2 mg vitamin B_1_, 3 mg vitamin B_6_.

In trial 1, 264 piglets (8.3 ± 1.22 kg) from 24 litters were weaned and allocated to 24 different pens in the same room. The room was previously presoaked, power washed, disinfected, and left to dry as per standard practice on the farm (Table 1). Pens were allocated to the three dietary treatments (Ct, Zn, or Ab; *n *= 8 pens per treatment), balanced by pen weight, and the pigs were fed the treatment for 1 week after weaning.

In trial 2, 180 piglets (8.5 ± 0.80 kg) from 18 litters were weaned and allocated to two rooms with nine pens each. Before introducing the pigs into the rooms, the rooms were cleaned with a substandard or optimal protocol. In the substandard protocol, presoaking and drying were not carried out, and in the optimal protocol, an extra step using detergent was added (Table 1). Within each room, pens were randomly allocated to one of the three dietary treatments, resulting in 3 pens per diet per room balanced by the pen weight (Ct, Zn, or Ab; *n *= 6 pens per treatment, 3 in each room).

In trial 3, 324 piglets (8.6 ± 1.26 kg) from 27 litters were weaned and allocated to three rooms with nine pens each. Before introducing the pigs into the rooms, the rooms were either not cleaned or cleaned with a substandard or standard protocol. For this trial, the substandard protocol did not include disinfection (Table 1). Within each room, the pens were randomly allocated to one of the three dietary treatments, resulting in 3 pens per diet per room, balanced by the pen weight (Ct, Zn, or Ab; *n *= 9 pens per treatment, 3 in each room).

### Sample collection, DNA extraction, and library preparation.

At 7 days postweaning, one random freshly voided fecal sample from one pig per pen replicate was collected, transferred to a 1.5-mL microcentrifuge tube and immediately stored at −80°C until processed. The samples were collected using a 140- by 7-mm conical steel spatula, avoiding the part in direct contact with the floor, and a new sterile spatula was used for each sample. DNA was extracted from the fecal samples using the QIAamp PowerFecal Pro DNA kit (Qiagen, Crawley, West Sussex, UK) following the manufacturer’s instructions. A fluorimeter (Qubit 3, Invitrogen) was used to determine the total DNA concentration. Paired-end sequencing libraries were prepared from the extracted DNA using the Nextera XT library preparation kit (Illumina Inc., San Diego, CA), followed by sequencing on the Illumina NextSeq 500 platform using high-output chemistry (2 × 150 bp) according to the manufacturer’s instructions. The size of the library for each sample was assessed using an Agilent Technology 2100 Bioanalyzer with a high-sensitivity DNA chip.

### Bioinformatics analysis.

Preprocessing of the raw reads by sequence quality and length was performed using PRINSEQ-Lite v0.20.4 (42). A mean quality score lower than Q25 in a 10-bp sliding window was the criteria used for trimming low-quality reads at the 3′ end. A minimum length of 150 bp was ensured for all reads. The clean Illumina sequences were screened against the pig reference genome (Sus scrofa UCSC) downloaded from Illumina iGenomes (https://support.illumina.com/sequencing/sequencing_software/igenome.html) to remove host reads using Bowtie2 v2.2 (43) with default values, in order to identify and remove host DNA sequences and reads derived from human DNA contamination. The unmapped reads were then used for the downstream analysis.

Read duplicates were removed using the Picard MarkDuplicates tool (https://broadinstitute.github.io/picard/) to create fastq files with unique reads only. Afterward, the reads were subjected to a further quality-filtering step. In brief, low-quality reads were trimmed using a modified version of the script trimBWAstyle.pl, which works directly from BAM files (TrimBWAstyle.usingBam.pl; https://github.com/genome/genome/blob/master/lib/perl/Genome/Site/TGI/Hmp/HmpSraProcess/trimBWAstyle.usingBam.pl). The script was used to trim bases with a quality value of three or lower. This threshold was chosen to delete all bases with an uncertain quality, as defined by Illumina’s EAMMS (end-anchored max scoring segments) filter.

The microbial composition analysis was carried out using the Kraken v2 species classifier (44). Functional profiles were assigned using SUPER-FOCUS (45). Metagenome assembly was performed using MEGAHIT (46).

### Statistical analysis.

Analyses were carried out in R v4.0.2 separately for each trial, studying the differences between diets and cleaning protocols. The average daily gain, average daily feed intake, and feed conversion rate were calculated, and differences between the treatments were analyzed using general linear models. The alpha and beta diversities were both computed at the species level using the R package vegan v2.5-7 (47). For estimation of the alpha diversity, the species richness, Chao1, Simpson, and Shannon indices of diversity were calculated. Statistical differences in the alpha diversity indexes were tested, after confirming their normal distribution, with ANOVA and pairwise compared using the Tukey test (car v3.0.10 [48], multcompView v0.1.8 [49], and lsmeans v2.30.0 [50] R packages) or tested using the Kruskal-Wallis test and pairwise tested using the Wilcoxon test (R package stats v4.0.2 [51]), when the data did not follow a normal distribution. The beta diversity was calculated and ordination of samples was performed using PCoA and nonmetric multidimensional scaling (NMDS) of the previously calculated Bray-Curtis distances between samples. The within-group dispersion was calculated using the betadisper function and tested with ANOVA. The separation between groups was tested with PERMANOVA (adonis 2 and pairwiseAdonis [52]). The factors and species influencing the ordination were assessed by fitting linear models to the ordination results (*envfit* function in the R package vegan). All *P* values were adjusted using the Benjamini-Hochberg (BH) approach. To fit species to the ordination space, the taxa were filtered, removing those species not present in at least 30% of samples and with less than a minimum threshold of total relative abundance of 0.005%. To produce a neat representation of fitted species on the ordination graphic, the species from trial 3 were subjected to a filter of 0.008% of total relative abundance.

Bacterial and function abundance analyses among treatments were performed using linear discriminant analysis (LDA) effect size (LEfSE) (53), with an alpha cutoff of 0.05 and an LDA threshold of 3, selecting the strictest analysis (all against all), and treatments as classes. Species explaining the differences between classes were determined by LEfSE using the Kruskal-Wallis test (*P < *0.05), followed by linear discriminant analysis. A cladogram of dietary treatment-associated taxa was produced using GraPhlAn (54). Core microbiomes of each dietary treatment group were calculated for a minimum threshold of 1% abundance in at least 70% of the samples of each group using the R package microbiome (55). Venn diagrams were built using the venn and get.venn.partitions functions in the gplots v3.1.1 and VennDiagram v1.6.20 packages, respectively (56, 57). Plots were built using ggplot2 v3.3.3 and pheatmap v1.0.12 (58, 59). Figures were produced using Inkscape v1.0.2 software (60).

### Data availability.

The full data sets have been submitted to the NCBI Sequence Read Archive (SRA) and are available under BioProject accession number PRJNA821641.
