# Supplementary material for: Using Shotgun Sequencing to Describe the Changes Induced by In-Feed Zinc Oxide and Apramycin in the Microbiomes of Pigs One Week Postweaning
Source: Microbiol Spectr. 2022 Aug 11;10(4):e01597-22. doi: 10.1128/spectrum.01597-22 (PMC9431492; doi:10.1128/spectrum.01597-22)
Supplement: Supplemental file 3 — Tables S1 and S2; Fig. S1 to S4. Download spectrum.01597-22-s0001.pdf, PDF file, 2.2 MB [file spectrum.01597-22-s0001.pdf]

Supplementary Table S1. Analysis (*envfit* function of Vegan) of the influence of different factors on the ordination of samples. Significance was established at α=0.05.

**Data**	**Factor**	**Ordination Method P. value (R2) Kraken2**	
		**PCoA**	**NMDS**
**Global**	Treatment	0.041 (0.071)	0.190 (0.043)
**Trial 1**	Treatment	0.111 (0.175)	0.444 (0.085)
**Trial 2**	Treatment	0.039 (0.209)	0.022 (0.221)
**Trial 3**	Treatment	0.007 (0.264)	0.104 (0.155)

Supplementary Table S2. Results of the permutation multivariate ANOVA test performed in the ordination analysis.

Data	Factor	P. value (R2)
**Global analysis**	Treatment	0.001 (0.109)
	Ct vs Ab	0.009 (0.054)
	Ct vs Zn	0.002 (0.121)
	Ab vs Zn	0.002 (0.074)
**Trial 1**	Treatment	0.068 (0.156)
	Ct vs Ab	0. 510 (0.059)
	Ct vs Zn	0.069 (0.181)
	Ab vs Zn	0.127 (0.133)
**Trial 2**	Cleaning	0.029 (0.088)
	Treatment	0.003 (0.169)
	Ct vs Ab	0.063 (0.118)
	Ct vs Zn	0.063 (0.139)
	Ab vs Zn	0.614 (0.076)
	Treatment:Cleaning	0.180 (0.095)
**Trial 3**	Cleaning	0.139 (0.097)
	Treatment	0.011 (0.148)
	Ct vs Ab	0.399 (0.064)
	Ct vs Zn	0.018 (0.168)
	Ab vs Zn	0.036 (0.121)
	Treatment:Cleaning	0.203 (0.167)

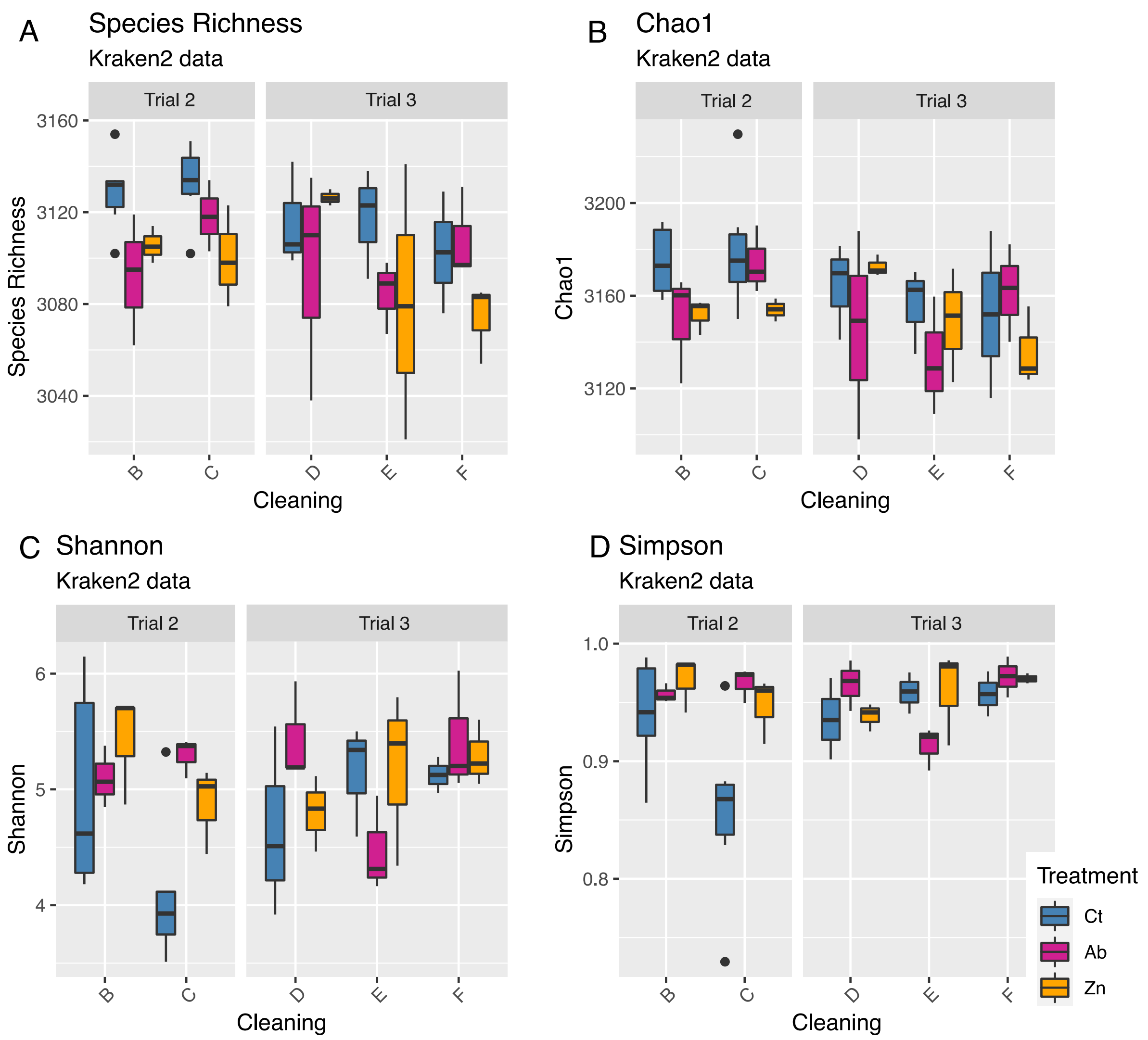

Supplementary Figure S1. Alpha diversity of different dietary treatments microbiomes, separated by cleaning procedures applied in trials 2 and 3, measured by Microbial richness (Species richness, Chao1 and Shannon) and evenness (Simpson index). The lower, medium, and upper horizontal box lines correspond to the first, second and third quartiles (the 25th, 50th and 75th percentiles). Upper and lower whiskers include the range of the upper and lower points within the 1.5 interquartile range. A) Species richness; B) Chao1 index; C) Shannon diversity index; D) Simpson's diversity index. *P < 0.05, **P < 0.01, and ***P < 0.001, respectively. Taxonomic identification of sequences was performed using Kraken2.

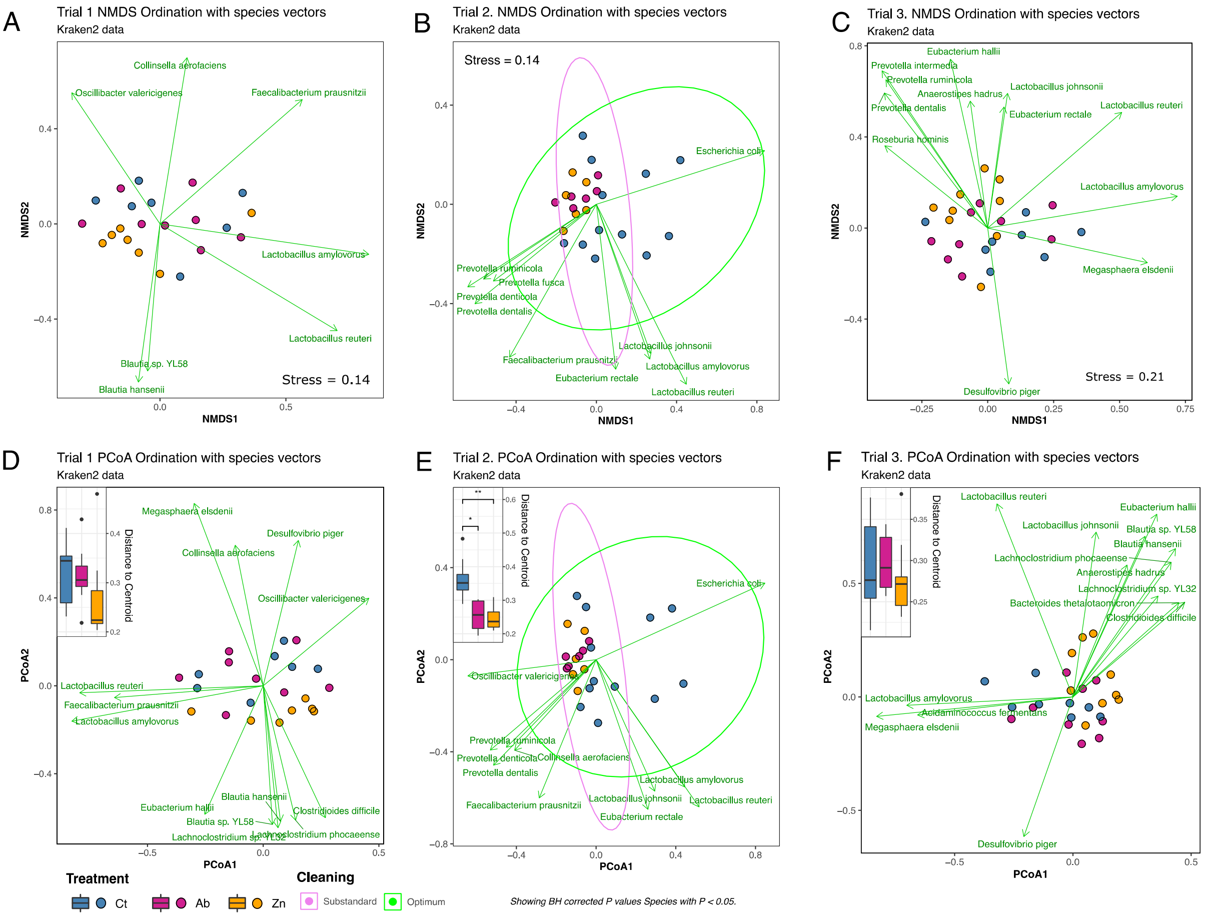

Supplementary figure S2. Ordination of microbiomes performed with NMDS, for trial 1, 2 and 3 (A, B, and C, respectively); and PCoA, for trials 1, 2 and 3 (D, E and F, respectively), with samples coloured by dietary treatment. Green arrows display the species returned by "envfit" model, influencing the ordination of samples (Arrows showing BH p.adjusted significant species; Arrows length shows the strength of each specie influencing the ordination of samples). Ellipses drawn on Figures B and E represent each cleaning group, with their shape being defined by the covariance within each group. Taxonomic identification of sequences was performed using Kraken2.

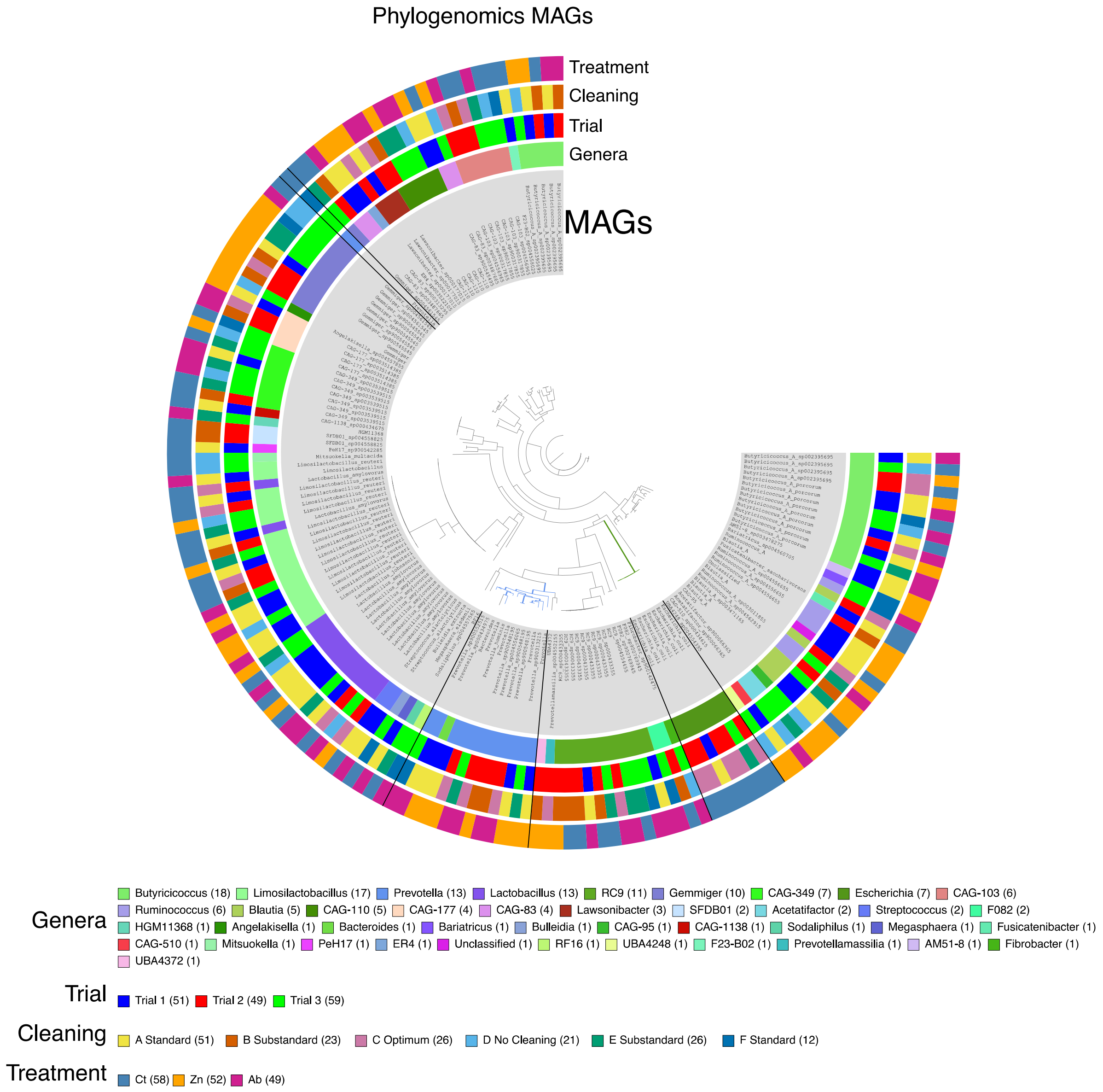

Supplementary Figure S3. Metagenome-Assembled genomes (MAGs) of samples using MEGAHIT. Seven genomes of *E. coli* found in Ct dietary treatment samples, as well as 12 genomes of *Prevotella* spp. in Ab and Zn, and 1 in Ct, are highlighted within 2 black bars going from inner to outer part of the circle.

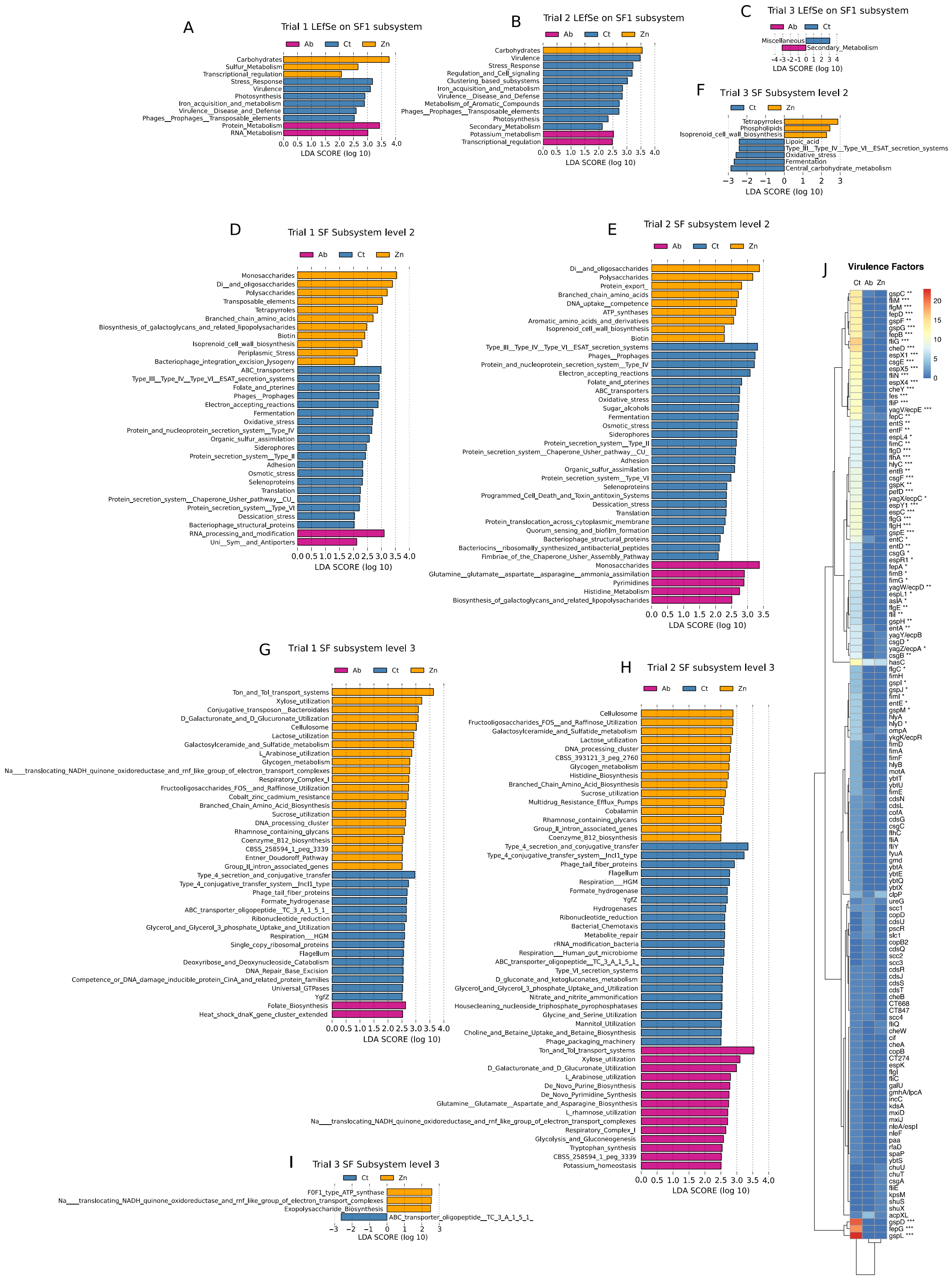

Supplementary Figure S4. Microbial functions differentially abundant identified for each dietary treatment group using LEfSe (A-I). (A-C) Results for the analysis performed for trial 1(A), 2 (B) and 3 (C) in level 1 of Super-Focus data. (D-F) Results for the analysis performed for trial 1(D), 2 (E) and 3 (F) in level 2 of Super Focus data. (G-I) Results for the analysis performed

for trial 1(G), 2 (H) and 3 (I) in level 3 of Super Focus data. (J) Heatmap showing virulence factors identified for each dietary treatment. *P < 0.05, **P < 0.01, and ***P < 0.001, respectively.
